# The Practical Aid of the Wassermann Reaction to the General Practitioner

**Published:** 1913-09

**Authors:** C. W. Gould

**Affiliations:** Atlanta, Ga.


### THE PRACTICAL AID OF THE WASSERMANN REACTION TO THE GENERAL PRACTITIONER.

By C. W. Gould, M. D., Atlanta, Ga.

Ever since the discovery of the complement fixation test in the French laboratories and afterward applied by Wassermann, Neisser and Bruck in 1906; the detection of the specific liptotropic substances formed in the blood by the action of the Spirochaetae Pallida, the medical fraternity has realized in a degree its importance.

However, we find that the full usefulness of the reaction has been rather slow in permeating through the profession.

Syphilis in its obscure forms has always been a bugbear in the minds of physicians. We never knew absolutely when we had it cured. We were rarely sure that the patient had the disease unless he had a clear-cut history, if he were one of the better class of patients, we dismissed the suspicion with a blush of shame at our effrontery in thinking such a thing possible in that family!

There is an old saying, "After everything else you have tried use Potassium Iodide," Osler said that "The result of treatment has a definite bearing on the diagnosis."

Syphilis is peculiarly insidious in its attacks upon humanity. A man may feel that he has led an irreproachable life in regard to sexual matters and yet find that because of some life, his pride of ancestry is turned to bitterness by the discovery that he has inherited along with his acres and pedigree, the disease of syphilis.

We have often found syphilis to be the cause of obscure symptoms which could not be accounted for on any rational basis.

A pure young lady kissed by her lover develops a chancre of the lip. It is mistaken for a fever blister and receives no attention until the secondary stage develops. A child kissed by its loving negro nurse; the public drinking cup, and the toilet, often are the beginning of syphilis, which because of the standing of the patient, or lack of history, or perhaps an attenuated virulency of the infecting spirochite produce symptoms which the practitioner puzzles over in vain until he turns to the Wassermann test.

Some physicians in their surprise and wonder at finding so many unexpected cases of syphilis avail themselves of its use very frequently.

Of course after finding that a patient has the disease, it is used as a check in the treatment. It is a strange fact that a case will sometimes be positive immediately after the administration of Salvarsan, which was negative before. This is accounted for by some as the breaking down by the treatment, of the lesions walled off from the circulation, thus allowing the products of the Spirochaetae to get into the circulation. Two negative Wassermanns were obtained only recently upon a patient, but because the Genito-Urinary Specialist was convinced of the presence of the disease, he went ahead and administered Neo-Salvarsan and had another Wassermann done the next day. The results was a strong positive test.

To illustrate how a very vague symptom suggesting a Wassermann test, the result of which was very gratifying to the patient and physician, I shall cite the case of a young man who was sent to me by a physician to try various laboratory tests, the Wassermann not being mentioned. Nothing was found in blood coniti, urine tests, etc. Finally because of a slight pain along the tibia, a Wassermann was made, his physician treating him with good results, after he had been to leaders of the profession in several of the large cities of the country.

A young lady who had suffered from recurrent severe attacks of asthma from babyhood, was tested because of the early manifestation of the disease might be a sign of hereditary syphilis. Her blood gave a positive test and after several treatments she had no sign of the disease.

A young man suffering from a high fever suggesting typhoid to the attending men, they asked me to do a blood culture. While they were obtaining the blood and putting it with culture medici, he spoke of having had very frequently "a rotten sore throat" or tonsilitis. A little blood was set aside and a Wassermann done without the patient or physician's knowledge. A very strong four-plus-positive reaction was obtained. The fact was communicated to the attending men with the result of a speedy cure. A similar case was of a lady of high social standing suffering from an acute attack of fever, which because of lack of confirmatory symptoms of the disease which one would naturally suspect, was puzzling the attending physician. However, he heard accidentally of the husband's having been to a skin and venereal specialist. A Wassermann was ordered with positive result, and a quick recovery after the medical treatment.

The above cases illustrate the advantages of the use of the Wassermann test when the diagnosis is very obscure. Of course when we have the primary lesion or have mucous patches where we may demonstrate the presence of the spirochetae, the Wassermann test is superfluous until after treatment has been instituted. Then it is of great use in checking results of the treatment.

It has a high place also in the diagnosis of the cause of Tabes Dorsalis, or of Paresis. If the spinal fluid in the early stages of these diseases is examined with a positive result, treatment at this stage gives good results.

Of the pitfalls which may give a wrong result is taking the blood of a patient who may be drinking alcoholics excessively. One is liable to get a negative result when, if the man's system were free from alcohol, he would give a strong positive. So it is well not to test the blood until several days have elapsed since the indulgence in alcohol.

Infecting the serum with certain Staphylococci and Streptococci at the time of taking it from the patient indicates the necessity of aseptic technic in obtaining the specimen.

The presence of malaria parasite often gives a misleading positive result also.

Although positive Wassermann is a good indication of syphilis, a negative reaction is not a sure indication of the absence of the disease. Repeated negative results after treatment is conclusive evidence, however, that the disease is cured.

The Wassermann reaction like any other sign ought to be taken as an important part of the symptom complex, and only that. All the facts of history and results of specific treatment must also be taken into consideration by the doctor in arriving at his diagnosis.

Conclusion:

1. The Wassermann test is of great use in confirming suspicion of a history of a primary or secondary lesion.

2. It is of great use in early diagnosis of Tabes or Paresis.

3. It is of great use in clearing up vague cases where one is trying various laboratory tests in an effort to locate the cause of the disease.

4. It is very valuable in checking up treatments.

5. Malaria must be ruled out before announcing that the patient has syphilis after a positive result.

6. One must obtain the blood in aseptic condition as soon as possible.

7. The drinking of alcohol vitiates the results of the Wassermann reaction.

653 Candler Building.

